# Economic evaluation of automated peritoneal dialysis among pediatric patients with end state kidney diseases in Thailand

**DOI:** 10.1038/s41598-025-00352-4

**Published:** 2025-05-25

**Authors:** Montira Assanatham, Sitaporn Youngkong, Montarat Thavorncharoensap, Anirut Pattaragarn, Usa Chaikledkaew

**Affiliations:** 1 Mahidol University Health Technology Assessment (MUHTA) Graduate Program, Mahidol University, Bangkok, Thailand; 2 Division of Nephrology, Department of Medicine, Faculty of Medicine Ramathibodi Hospital, Mahidol University, Bangkok, Thailand; 3 Social and Administrative Pharmacy Division, Department of Pharmacy, Faculty of Pharmacy, Mahidol University, Bangkok, Thailand; 4 Division of Nephrology, Department of Pediatrics, Faculty of Medicine Siriraj Hospital, Mahidol University, Bangkok, Thailand

**Keywords:** Continuous ambulatory peritoneal dialysis, Automated peritoneal dialysis, Cost-utility analysis, End-stage kidney disease, Thailand, Kidney diseases, Renal replacement therapy, Health care, Medical research

## Abstract

Currently, due to the absence of economic evaluation information, automated peritoneal dialysis (APD) is not included in Thailand’s Universal Health Coverage (UHC) benefit package. Therefore, we aimed to assess the cost-utility and budget impact of APD and continuous ambulatory peritoneal dialysis (CAPD) in pediatric end-stage kidney disease (ESKD) patients. A Markov model was applied to compare lifetime costs and health outcomes based on a social perspective using cost, utility, and transitional probability data from literature reviews. The results were presented as the incremental cost-effectiveness ratio (ICER). Moreover, one-way and probabilistic sensitivity analyses were performed to evaluate the uncertainty of all parameters. From a social perspective, patients receiving APD had higher total lifetime cost (14,791,473 baht) than those receiving CAPD (13,380,356 baht), but fewer life years (APD = 18.39, CAPD = 18.44) and higher quality adjusted life years (QALY) (APD = 16.31 and CAPD 15.65). At the societal willingness to pay in Thailand equal to 160,000 baht per QALY gained, APD would not be cost-effective (ICER = 3,063,598 baht per QALY gained). The inclusion of APD can result in an increase in annual budget of 54 million baht. Therefore, this study would help inform policy decisions regarding the inclusion of APD for pediatric with ESKD in the UHC benefit package.

**Supplementary Information:**

The online version contains supplementary material available at 10.1038/s41598-025-00352-4.

## Introduction

The incidence of end-stage kidney disease (ESKD) and kidney replacement therapy varies according to genetic, environmental and economic factors^1,2^. A study by Harambat J et al. found that the median incidence of chronic kidney disease (CKD) worldwide in children and adolescents under 19 years is 9 people (range 4–18 people) per one million people in the same age group^1^. According to the annual report on kidney replacement therapy in Thailand, there were 531 pediatric patients with CKD registered to receive kidney replacement therapy, with 36% receiving hemodialysis (HD), 58% undergoing peritoneal dialysis (PD), and 100% had kidney transplants^3^.

Kidney transplantation is the most appropriate treatment for patients with end-stage renal failure, as kidney transplant patients experience a higher quality of life and survival rate compared to those receiving other forms of kidney replacement therapy^4^. However, if the patients face limitations preventing them from undergoing a kidney transplant, they will receive other kidney replacement therapies, such as PD or HD in children. The decision to choose a specific kidney replacement therapy varies for each patient and family, and is influenced by factors such as age, lifestyle, and family preferences, and the medical team’s assessment of patient’s and family’s ability to comply with treatment^5^.

PD is a more common option for children, particularly for infants and young children. While survival rates for PD and HD are similar, PD offers several advantages, particularly in preserving remaining kidney function, or residual renal function (RRF), better than HD. Additionally, PD provides more flexibility in managing water and food restrictions, which is especially beneficial for infants and young children. These patients rely on milk primary source of nutrition, making it difficult to limit water intake. PD also eliminates the need for vascular access, a challenge in HD, particularly in younger children^6,7^.

PD can be categorized into two main methods: manual PD, where patients change the dialysis solution themselves (also known as continuous ambulatory peritoneal dialysis or CAPD) and automated PD (APD), where a machine assists in the fluid exchange process. In Thailand, CAPD is currently included in the Universal Health Coverage (UHC) benefit package, while APD is more common in countries with fewer cost constraints, such as the United States, where its use among infants, children, and adolescents is as high as 82.9%^8,9^.

APD is popular for several reasons. It uses a machine to change the dialysis fluid at night while the patients sleep, allowing the patients to continue their daily activities, such as attending school, without nightly intermittent peritoneal dialysis (NIPD) or continuous cyclic peritoneal dialysis (CCPD)^8^. It also reduces common discomforts such as constipation and decrease the risk of hernias^8^. Moreover, APD has a lower incidence of peritoneal infections compared to CAPD, as it involves fewer dialysis bag changes^8^, thus reducing the risk of contamination of dialysis solution per cycle. APD is also more flexible, as it allows for adjustments in the number of cycles and the amount of dialysis solution per cycle, tailored to the patient’s age, body size, and peritoneal characteristics, making it a more convenient option than CAPD^10^.

However, APD also has disadvantages compared to CAPD. For instance, the increased number of fluid exchanges in APD can result in a higher risk of malnutrition due to greater loss of protein and amino acids^11^. CAPD may also better preserve residual kidney function compared to APD^12^. Additionally, the noise from the machine during nighttime APD treatments may disturb the sleep of both the patient and the caregiver^12^. Despite these challenges, APD offers potential benefits in pediatric patients with ESKD, although it comes at a higher cost than CAPD^12,13^. Currently, there has been no cost-utility analysis comparing APD with CAPD in pediatric patients. This study aimed to evaluate the cost-utility and budget impact of APD compared to CAPD in pediatric ESKD patients from a societal perspective. The findings would provide crucial information to guide policymakers in making decisions about supporting APD as a kidney replacement therapy option for pediatric ESKD patients.

## Methods

### Study design

A cost-utility analysis using a Markov model was applied to compare costs and health outcomes of APD with CAPD over a lifetime horizon, with a one-year cycle length, from a societal perspective. This method allows for the estimation of long-term costs and quality-adjusted life years (QALYs) associated with both treatment options. Additionally, a budget impact analysis was conducted to estimate the financial burden if APD were to be included in the UHC benefit package from a governmental perspective. To further validate the model structure and all parameters, face validity checks were conducted through consultation meetings with nephrologists from the 11 hospitals participating in a randomized controlled trail (RCT) conducted in Thailand^14^, as well as with two health economists. This validation process strengthens the reliability of model structure and all parameters used in our study.

### Target population

The target population for this study comprised all ESKD patients under 18 years of age. The Markov model started at an age of one year, with a lifetime time horizon applied for the analysis. Ethics approval for the study was obtained from the Central Research Ethics Committee (CREC) under the approval number CREC083/62BRm. Informed consent was waived by the ethics committees, as the data were obtained from published studies. All methods were carried out in accordance with International Guidelines for Human Research Protection such as the Declaration of Helsinki, Belmont Report, CIOMS Guidelines and International Conference on Harmonization in Good Clinical Practice (ICH-GCP).

### Model structure

**Fig. 1:**
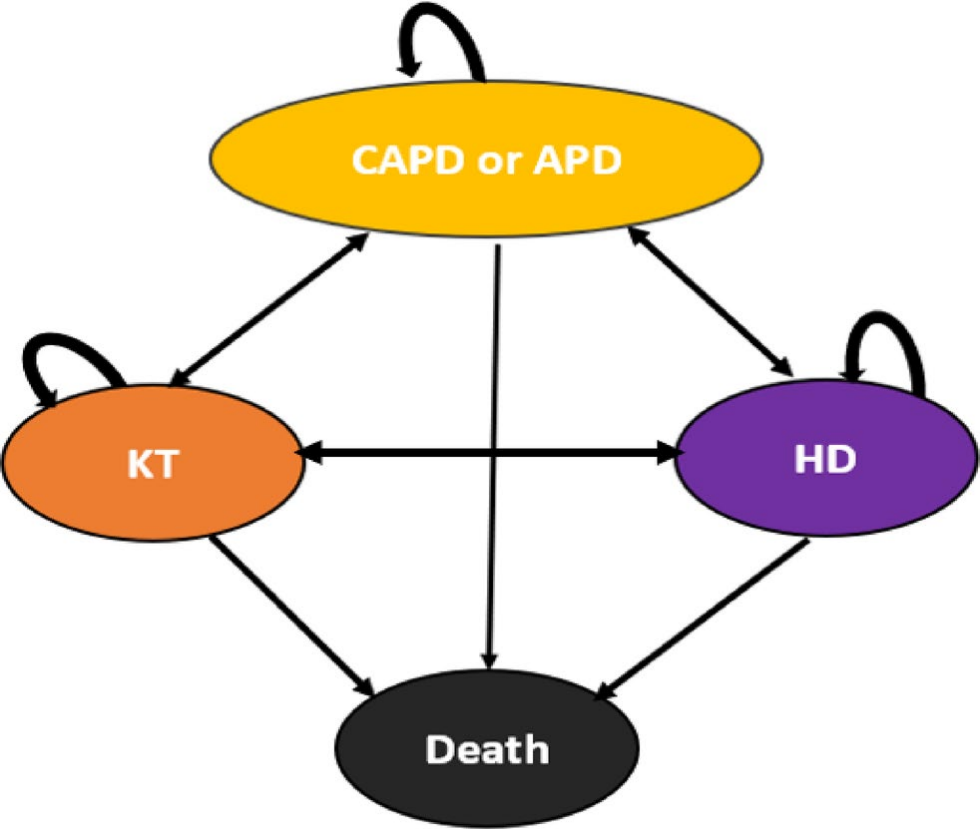
Markov model. *CAPD* continuous ambulatory peritoneal dialysis; *APD* automated peritoneal dialysis; *KT* kidney transplantation; *HD* hemodialysis.

As depicted in Fig. 1, pediatric ESKD patients had the option to receive either APD or CAPD, which are the treatment options analyzed in this study. Regardless of which dialysis method was chosen, the patients entered a Markov model consisting of five health states: (1) receiving APD or CAPD (2) receiving HD, (3) kidney transplantation (KT), and (4) death. Arrows indicate the probabilities of transitioning from one state to another. Patients receiving APD or CAPD were more likely to be transferred to HD or to undergo KT. In cases of failure, there was a possibility of being transferred back to APD or CAPD, or to HD dialysis. Additionally, in each health state i.e., APD, CAPD, HD, or KT, the patient could either remain in the current state or transition to the deceased state.

### Model assumptions

Several assumptions were made in this mode. First, it was assumed that no patient would switch between APD to CAPD or vice versa. Second, if patients who received HD or KT experienced failure, they had the option to return to their initial dialysis, either APD or CAPD. Third, since there was no available data on the probability of transitioning from APD to KT, it was assumed to be the same as the probability of transitioning from CAPD to KT. Lastly, the transitional probabilities from the 10th year onward were assumed to remain constant, using the probabilities from the 10th year.

### Model parameters

#### Transitional probabilities

Supplementary Table 1 shows all variables used in the model.

Transitional probabilities for APD to HD, PD to KT, APD to death, CAPD to HD, CAPD to death, HD to PD, HD to APD, HD to death in the first year were sourced from a previous study^14^. This study was an open-labelled, multi-center RCT conducted across 11 public tertiary care hospitals throughout Thailand^14^. The RCT investigated the clinical outcomes of ESKD patients aged 1–18 years, comparing those receiving APD (30 patients) compared to those receiving CAPD (30 patients)^14^. Additionally, these transitional probabilities from the second year onward were obtained from a prospective cohort study of 596 pediatric patients receiving kidney replacement therapy during three years of follow-up in Asia, a similar setting to Thailand^15^. To derive these transitional probabilities, we first obtained the patient survival probabilities (S(t)) for years one to three from the cohort study^15^. We then used the observed survival rate decrease between years two and three to extrapolate these probabilities for years four to ten. The survival probability for each cycle (i.e., one year) (S(u)) was calculated by dividing the survival probability at year t by the survival probability of the previous year (S(t)/S(t−1)). The probability of failure or event of interest (tp(t)) for each cycle was calculated as tp(t) = (1−S(u)), and the annual failure rate or event of interest was determined using this formula: −ln(1−tp(t))/cycle). Finally, the annual transitional probability of failure or event of interest (tp(u)) was calculated as 1−exp (−tp(u)). Probabilities for transitions from KT to HD, PD, APD or death were retrieved from published studies^16–18^. Additionally, transitional probabilities of death by age were obtained from the age-specific mortality rate for Thai population^19^.

### Costs

This study considered both direct medical and direct non-medical costs from a societal perspective. Direct medical costs included expenses for dialysis, outpatient visits, and inpatient visits related to APD, CAPD, HD, or KT, while direct non-medical costs covered travel expenses for treatment, food, caregiver wages, and caregiver opportunity costs. Both types of costs were sourced from a previous open-labeled, multi-center RCT study conducted in Thailand^14^, which reflects routine clinical care costs, rather than additional costs driven by research protocols. This ensures that the costs used in the analysis are representative of real-world practice.

Indirect costs, such as opportunity costs for treatment time or losses from illness or patient death, were excluded to avoid double-counting. All historical cost data were adjusted to 2022 values using the Consumer Price Index from the Ministry of Commerce^20^. Future costs and health outcomes across different time periods were discounted to present values at a rate of 3%, following the recommendations outlined in Thailand’s Health Technology Assessment (HTA) guidelines^21^.

### Utility

Utility data for this study were derived from a recent utility study^22^ based on the previously mentioned RCT conducted in Thailand^14^. This study collected utility data from 60 patients aged 1–18 years, including 30 on CAPD and 30 on APD using the EuroQoL-5D-5L (EQ-5D-5L) instrument, with responses provided by their parents. The utility score of CAPD (0.8900) was lower compared to that of APD (0.9400). Moreover, the utility values of HD and KT were sourced from published studies^23,24^.

### Result presentation

The total costs, life years (LYs), and QALYs associated with the two options (APD or CAPD) were calculated. The incremental cost-effectiveness ratio (ICER) was determined by dividing the incremental cost by the incremental QALYs associated with APD compared to CAPD. The ICER was presented as the cost (in baht) per QALY gained. The cost-effectiveness analysis was conducted using a willingness-to-pay (WTP) threshold of 160,000 baht per QALY gained, in accordance with the guidelines from the Subcommittee for the Development of the National List of Essential Medicines (NLEM) (16).

### Uncertainty analysis

To evaluate the model’s sensitivity to individual input parameters, we conducted both one-way and probabilistic sensitivity analyses (PSA). In the one-way sensitivity analysis, each input parameter was adjusted within its 95% confidence interval (CI), and the resulting range of ICER values was visually represented using a Tornado diagram. Furthermore, PSA was used to assess the uncertainty across all parameters simultaneously through a Monte Carlo simulation consisting of 1000 iterations. A cost-effectiveness acceptability curve (CEAC) was then used to determine the probability of each option being considered cost-effective based on the societal Thai WTP threshold.

### Budget impact analysis

A budget impact analysis was conducted from the government’s perspective to assess the financial feasibility of providing APD to pediatric patients with ESKD. To evaluate the budget impact over ten consecutive fiscal years, a Markov model-based approach was employed to estimate the financial consequences of implementing APD for pediatric ESKD patients in Thailand.

The overall budget was calculated by multiplying the estimated number of pediatric ESKD patients requiring PD by the annual direct medical costs per patient for CAPD (433,641 baht) and APD (496,836 baht). The incremental budget for APD and CAPD, the difference in the total budget between APD and CAPD, and the net benefit impact (NBI) per patient were presented. The estimated number of pediatric ESKD patients was derived from data on the prevalence and incidence of all ESKD patients in Thailand requiring renal replacement therapy (RRT) across all age groups from 2016 to 2020. These figures were multiplied by the proportion of pediatric patients requiring RRT (0.004) and the probability of receiving PD (0.76)^3^. The total number of pediatric patients calculated from 2020 prevalence and incidence data was then used to estimate the number of pediatric patients requiring PD from 2021 to 2032, as shown in Supplementary Table 2.

Then, the number of patients in 2021 was calculated as the sum of the 2020 pediatric ESKD patient population and 61 incident cases. This process was repeated for subsequent years. The number of prevalent and incident cases requiring PD (C) was adjusted by subtracting the number of patients who received KT (probability = 0.079), those receiving HD (probability = 0.031), and those who died (probability = 0.023). The estimated number of remaining pediatric patients requiring PD is shown in Supplementary Table 3. In addition, if APD was adopted into the UHC benefit package, all children would receive the APD modality in the first year of adoption. Therefore, a 100% uptake rate for the BIA was applied in this study.

## Results

### Cost-utility analysis

Table 1 presents the results of the cost-utility analysis, including total costs, overall LYs, total QALYs, and the ICER for APD in comparison to CAPD. From a societal perspective, the APD group exhibited a higher total lifetime cost of 14,791,473 baht compared to 13,380,356 baht for the CAPD group. Patients in the APD group had a total of 18.39 LYs and 16.31 QALYs, while those in the CAPD group had 18.44 LYs and 15.65 QALYs. Notably, the APD group had 0.05 fewer LYs but 0.46 additional QALYs compared to the CAPD group. From a social perspective, the ICER for the APD group was 3,063,598 baht per QALY gained compared to CAPD group.

**Table 1:** Cost-utility analysis results.

Results	CAPD	APD
Total costs (baht)	13,380,356	14,791,473
Total life years	18.44	18.39
Total QALYs	15.85	16.31
Incremental costs		1,411,107
Incremental LYs		−0.05
Incremental QALYs		0.46
ICER (baht/LY gained)		−25,984,548 (Dominated)
ICER (baht/QALY gained)		3,063,598

### Uncertainty analysis

**Fig. 2:**
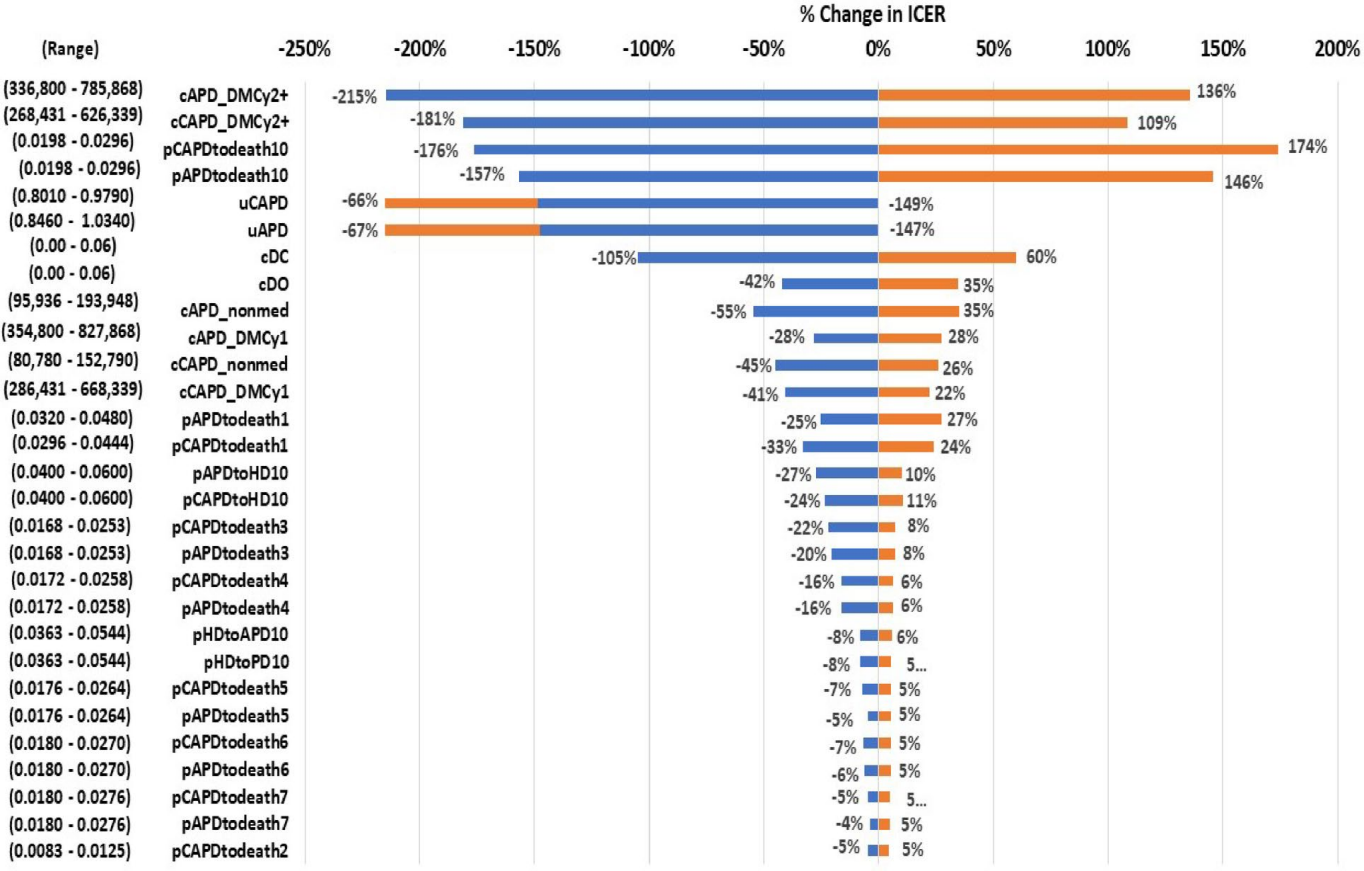
displays the results of the one-way sensitivity analysis using a Tornado diagram. The analysis revealed that the variable causing the greatest change in the ICER value was the direct medical costs of APD patients in the 2nd year, followed by the direct medical costs of CAPD patients in the 2nd year, the probability of transitioning from CAPD to death in the 10th year, the probability of transitioning from APD to death in the 10th year, and the utility values of both CAPD and APD patients, respectively.

Figure 2 Tornado diagram. *ICER* incremental cost-effectiveness ratio. *cAPD_DMCy2 +* Total direct medical costs of APD from year 2 onward; *cCAPD_DMCy2 +* Total direct medical costs of CAPD from year 2 onward; *pCAPDtodeath10* Probability of CAPD to death in year 10; *pAPDtodeath10* Probability of APD to death in year 10; *uCAPD* Utility of CAPD; *uAPD* Utility of APD; *cDC* Distcount rate for costs; *cDO* Distcount rate for outcomes; *cAPD_nonmed* Direct non-medical costs for APD; *cAPD_DMCy1* Total direct medical costs of APD in year 1; *cCAPD_nonmed* Total direct non-medical costs for CAPD; *cCAPD_DMCy1* Direct medical costs of CAPD in year 1; *pAPDtodeath1* Probability of APD to death in year 1; *pCAPDtodeath1* Probability of CAPD to death in year 1; *pAPDtoHD10* Probability of APD to HD in year 10; *pCAPDtoHD10* Probability of CAPD to HD in year 10; *pCAPDtodeath3* Probability of CAPD to death in year 3; *pAPDtodeath3* Probability of APD to death in year 3; *pCAPDtodeath4* Probability of CAPD to death in year 4; *pAPDtodeath4* Probability of APD to death in year 4; *pHDtoAPD10* Probability of HD to APD in year 10; *pHDtoPD10* Probability of HD to PD in year 10; *pCAPDtodeath5* Probability of CAPD to death in year 5; *pAPDtodeath5* Probability of APD to death in year 5; *pCAPDtodeath6* Probability of CAPD to death in year 6; *pAPDtodeath6* Probability of APD to death in year 6; *pCAPDtodeath7* Probability of CAPD to death in year 7; *pAPDtodeath7* Probability of APD to death in year 7; *pCAPDtodeath2* Probability of CAPD to death in year 2.

According to Supplementary Table 1, transitional probabilities from year 2 to year 10 were identical regardless of whether the APD or CAPD modality was used for any health state. Thus, only the first year affected the results. Scenario analysis was conducted using the transitional probabilities from the first year onward. Based on a societal perspective, the APD group incurred a higher total lifetime cost (11,347,593 baht) compared to the CAPD group (10,020,062 baht). Pediatric ESKD patients in the APD group had a total of 14.90 LYs and 13.59 QALYs, while those in the CAPD group had 15.37 LYs and 13.44 QALYs. As a result, the APD group had 0.47 fewer LYs but 0.15 additional QALYs compared to the CAPD group. The ICER for the APD group was 8,816,394 baht per QALY gained compared to CAPD group.

**Fig. 3:**
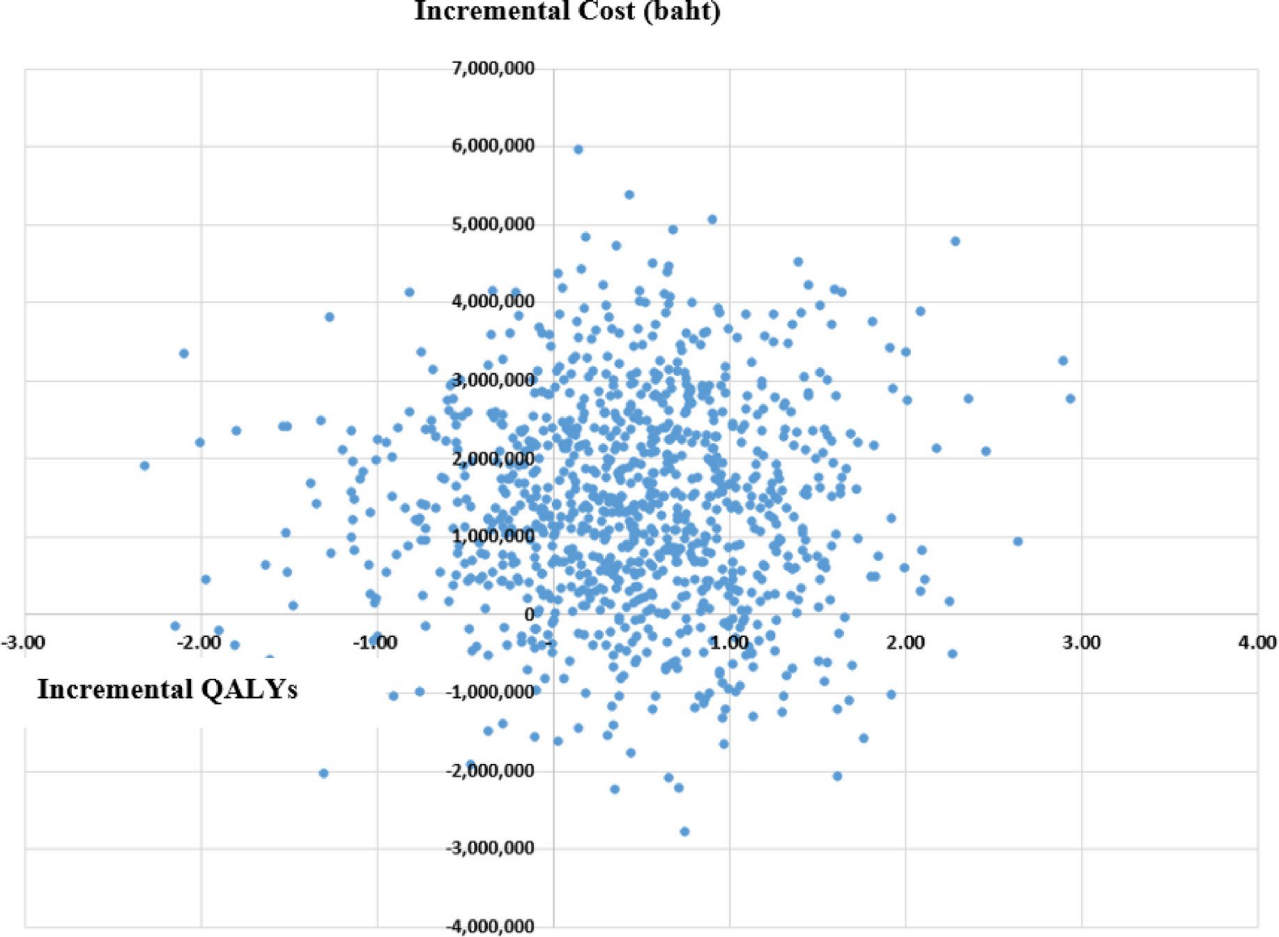
Cost-effectiveness plane. *QALY* quality adjusted life year.

PSA using the cost-effectiveness plane illustrates the differences in costs (incremental cost) and QALYs (incremental QALYs) between dialysis with APD and CAPD. The results showed that APD dialysis resulted in higher QALYs but also increased costs compared to CAPD from a societal perspective (Fig. 3).

**Fig. 4:**
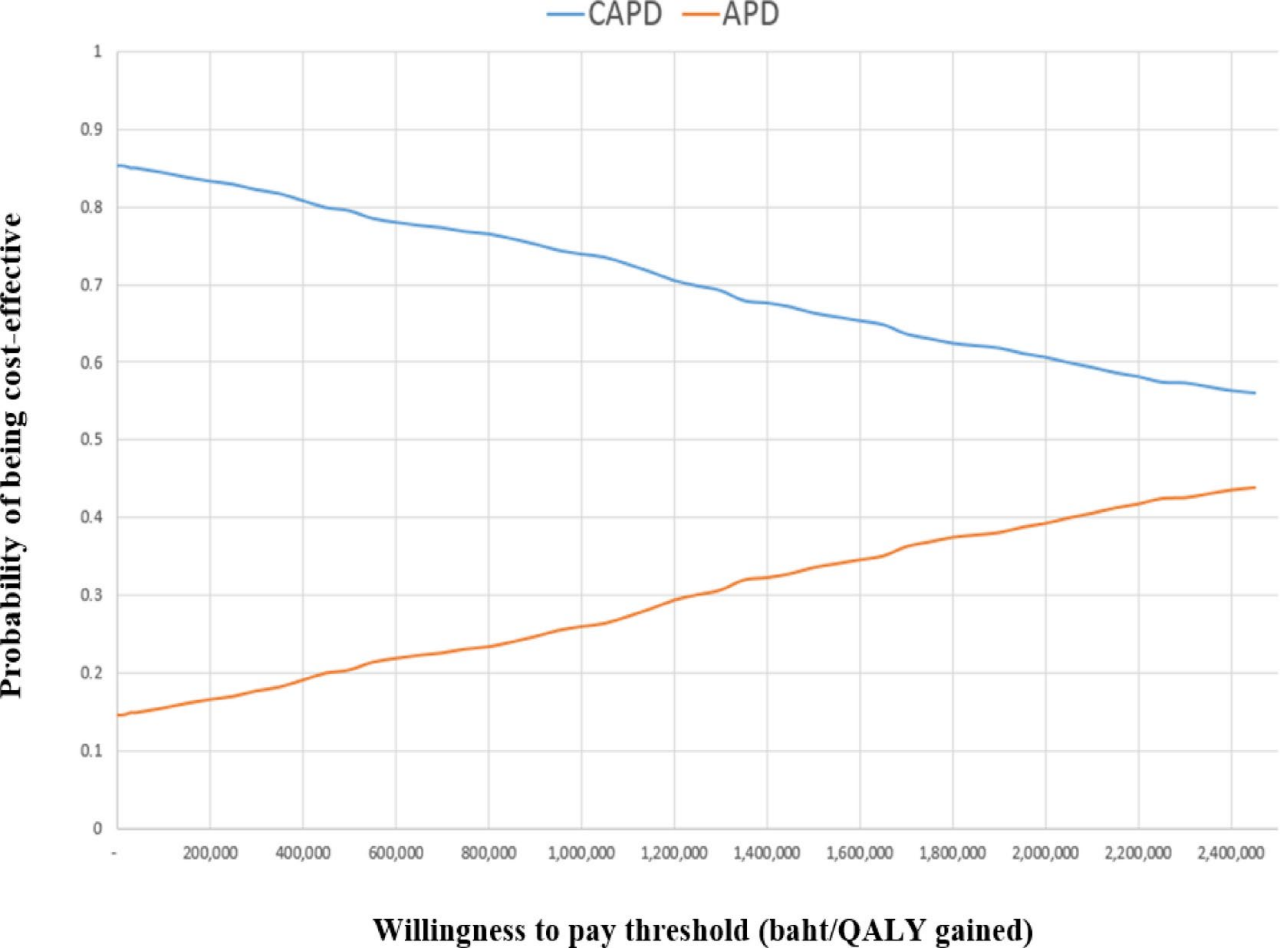
Cost-effectiveness acceptability curve. *CAPD* continuous ambulatory peritoneal dialysis; *APD* automated peritoneal dialysis; *QALY* quality adjusted life year.

Furthermore, by running 1000 iterations with randomized variables, the results were presented as CEAC. These curves graphically display the relationship between societal WTP at various levels (on the X-axis) and the probability that dialysis will be considered cost-effective (on the Y-axis). According to Thailand’s HTA guidelines, the maximum WTP threshold was set at 160,000 baht per QALY gained. The results showed that when the WTP threshold is set at 0, dialysis using the CAPD modality has a significant probability of being considered cost-effective, with an 85% likelihood from a societal perspective (as shown in Fig. 4), compared to APD.

### Budget impact analysis

The results presented in Table 2 indicated that the budget impact analysis of APD compared to CAPD for the years 2023 to 2032 yielded an average of approximately 853 ESKD patients aged 1–18 years per year. The average total budget for these patients, including the costs of dialysis, was estimated at 370 million baht for CAPD and 424 million baht for APD. Therefore, the budget impact of using APD instead of CAPD would result in an average annual budget increase of 54 million baht, with an average NBI per patient of 63,202 baht.

**Table 2:** Budget impact analysis results of APD compared with CAPD.

Year	Number of pediatric patients with ESKD	Total budget of CAPD (million baht)	Total budget of APD (million baht)	Incremental budget of APD compared with CAPD (million baht)	Net benefit impact (NBI) per patient (baht)
2023	618	268	307	39	63,107
2024	671	291	333	42	62,593
2025	724	314	360	46	63,536
2026	778	337	387	49	62,982
2027	831	360	413	53	63,779
2028	884	383	439	56	63,348
2029	937	406	466	59	62,967
2030	990	429	492	63	63,636
2031	1043	452	518	66	63,279
2032	1051	456	522	66	62,797
Average	853	370	424	54	63,202

## Discussion

This study represents the first attempt to evaluate the cost-utility of APD compared to CAPD in pediatric patients with ESKD in Thailand using a Markov model. Costs and utility data were obtained from a previous RCT study conducted in Thailand^14^, along with a review of relevant published literatures from both Thailand and international sources. The results indicated that in accordance with Thailand’s HTA guidelines, which set the WTP threshold at 160,000 baht per QALY gained, APD is not currently cost-effective (ICER = 3,063,598 baht per QALY gained) based on a societal perspective. Additionally, the adoption of APD is projected to increase the annual budget by an average of 54 million baht compared to CAPD, with an average NBI per patient of 63,202 baht. Notably, the most influential variables affecting ICER values included the direct medical costs of APD and CAPD in the second year, as well as the probabilities of transitioning from dialysis (CAPD or APD) to death in the tenth year. Furthermore, utility values for patients undergoing CAPD and APD were key determinants.

According to the base-case results, APD resulted in a slightly shorter LYs (−0.05) compared to the CAPD. The transitional probabilities for year one were derived from the referenced RCT^14^, which had a relatively sample size of 30 patients per modality. This limited sample size may have introduced variability in survival estimates, potentially affecting LY calculations. Nonetheless, the baseline characteristics of pediatric ESKD patients in the RCT showed no statistically significant differences in age, gender, education, hospital settings, time since diagnosis, time on dialysis, or comorbidities^14^. Further research with larger sample sizes and more diverse data sources is needed to refine these estimates and strengthen the evidence base for policy decision-making.

For transitional probabilities from years two to ten, data were obtained from a published cohort study conducted in Asia^15^, which included Malaysia, the Philippines, and Singapore, but not Thailand. While this study provides the best available evidence for estimating transitional probabilities from a comparable context, differences in age distribution, comorbidities, dialysis modalities, healthcare access, and treatment practices between these countries and Thailand may affect the accuracy of the estimated probabilities. This limitation should be considered when interpreting the results, and further research using Thai-specific data would enhance the accuracy of future estimations. To address this limitation, scenario analyses were conducted using year-one transitional probabilities applied across the entire model horizon. These analyses aimed to assess the robustness of the results under various assumptions better reflecting the characteristics of the Thai RCT population. The scenario analysis indicated that the APD group experienced fewer LYs (−0.47) but more QALYs (0.15) and higher costs, reinforcing a conclusion that APD is not cost-effective compared to CAPD. Compared to the incremental LYs and QALYs in the base-case analysis (−0.05 LYs and 0.46 QALYs), those from the scenario analysis were approximately three times lower. This difference occurred because the probabilities of death for both APD and CAPD in year one were higher than in subsequent years. By applying the higher year-one mortality rates across the entire model horizon, patients in both groups experienced earlier mortality, resulting in shorter survival times (APD = 14.90 LYs and 13.59 QALYs; CAPD = 15.37 LYs and 13.44 QALYs) and reduced differences in life years and QALYs between APD and CAPD, compared to the base-case analysis (APD = 18.39 LYs and 16.31 QALYs; CAPD = 18.44 LYs and 15.85 QALYs), where mortality risks decreased over time.

The limitations of this study should be acknowledged. First, certain costs associated with APD usage, such as electricity expenses for operating the APD machine, were excluded. As a result, only the cost of the APD solution was included in the calculations, potentially underestimating the true cost of APD. Future studies should incorporate these additional costs for a more comprehensive cost assessment. Second, due to the lack of utility data for pediatric patients undergoing HD and KT, utility values were derived from adult populations. The utility value for HD was obtained from a cross-sectional survey of 367 adult patients undergoing HD at 11 hospitals across various regions of Thailand, using the EQ-5D-5L instrument^23^. Similarly, we used utility values of KT from a UK study because there was no available Thai-specific utility data for pediatric patients undergoing KT at the time of the analysis. Since no Thai study specifically reported KT utilities, we chose the UK study as it provided robust, well-conducted, and widely accepted estimates^24^. The utility value for KT was sourced from data on 512 adult patients who underwent KT in the United Kingdom between November 1, 2011, and September 30, 2013, also using the EQ-5D-5L instrument^24^. These adult-derived utility values may not fully reflect the health-related quality of life of pediatric ESKD patients. Ideally, country-specific data would be used, but in the absence of such data, using international sources like the UK study is a common and accepted approach, especially if the healthcare context and patient characteristics are reasonably comparable. Therefore, future studies should focus on obtaining Thai pediatric-specific utility values of HD and KT to enhance the accuracy of cost-utility assessments in this population. Moreover, we did not account for the increase in utility associated with KT during the second-year post-transplant. Future studies should consider this aspect, as patients typically experience improved utility in the second year following kidney transplantation.

Dialysis for pediatric patients with ESKD has significant social implications that differ from those experienced by adults. The burden of dialysis extends beyond the patients themselves, affecting their caregivers and families. Pediatric patients often impose a considerable burden on their families compared to adult patients. APD offers advantages in this context by enabling pediatric patients to maintain their daily routines, including attending school and participating in social activities, without relying on home-based dialysis. This fosters a sense of normalcy in their lives, allowing them to engage in social and educational activities with their peers. Simultaneously, caregivers, particularly those of working age, can seek employment outside the home, ensuring the financial stability of their families.

However, it is crucial to recognize that dialysis represents a long-term commitment for pediatric patients awaiting kidney transplants. Despite the advantages of APD, effective long-term care remains necessary, including timely access to kidney transplants. Given the challenges associated with timely transplants for pediatric patients, the government should provide social support for these children and their families. This support should encompass psychological rehabilitation, guidance in preparing for kidney transplants, and financial assistance for both medical and non-medical expenses. Additionally, APD support should include education on the proper use and maintenance of APD machines.

Given Thailand’s WTP threshold of 160,000 baht per QALY gained, APD is not currently cost-effective compared to CAPD for pediatric patients with ESKD. However, the study also underscores the significant benefits of APD in enabling pediatric patients and their caregivers to maintain their daily routines, including education and employment. This results in a slight budget impact increase, averaging 54 million baht per year. Therefore, it is recommended that APD modality be included in the UHC benefit package. Additionally, the government should implement social support measures for pediatric patients and their families, including psychological rehabilitation, transplant preparation guidance, financial assistance, and APD education and maintenance support.

## Electronic supplementary material

Below is the link to the electronic supplementary material.

Supplementary Material 1

Supplementary Material 2

Supplementary Material 3

## Data Availability

Due to patient confidentiality concerns, we are unable to publicly share the raw data used in this study, as they pertain to Thai ESKD patients under 18 years of age. However, datasets related to costs and utility can be requested from the Central Research Ethics Committee (CREC) under approval number CREC083/62BRm. Qualified researchers can apply for access by contacting the CREC via official@crecthailand.org and providing a formal request outlining the intended use of the data. Regarding the training, validation, and test datasets, these are not publicly available but may be obtained from the corresponding author upon reasonable request, subject to ethical approval and data-sharing agreements.

## Code availability

The code used for data generation and analysis can be accessed and is available upon request. Additionally, data analysis was performed using Microsoft Excel software version 2021 (Microsoft Corporation, Redmond, WA, USA). The specific variables and parameters used in the analysis are presented in Supplementary Table 1.
