# Supplementary material for: Economic evaluation of automated peritoneal dialysis among pediatric patients with end state kidney diseases in Thailand
Source: Sci Rep. 2025 May 25;15:18259. doi: 10.1038/s41598-025-00352-4 (PMC12104364; doi:10.1038/s41598-025-00352-4)
Supplement: Supplementary file 1 — Supplementary Material 1 [file 41598_2025_352_MOESM1_ESM.docx]

**Supplementary Table 3** Estimated number of ESKD pediatric patients requiring PD from 2021 to 2032

| **Year** | **Number of incident and prevalent pediatric ESKD cases requiring PD (C)** | **Number of pediatric ESKD patients receiving KT (C*0.072)** | **Number of pediatric ESKD patients receiving HD (C*0.031)** | **Number of deaths (C*0.023)** | **Number of remaining pediatric ESKD patients requiring PD** |
| --- | --- | --- | --- | --- | --- |
| 2021 | 585 | 42 | 18 | 13 | 512 |
| 2022 | 646 | 46 | 20 | 15 | 565 |
| 2023 | 707 | 51 | 22 | 16 | 618 |
| 2024 | 768 | 55 | 23 | 18 | 671 |
| 2025 | 828 | 60 | 25 | 19 | 724 |
| 2026 | 889 | 64 | 27 | 20 | 778 |
| 2027 | 950 | 68 | 29 | 22 | 831 |
| 2028 | 1,011 | 73 | 31 | 23 | 884 |
| 2029 | 1,071 | 77 | 33 | 25 | 937 |
| 2030 | 1,132 | 81 | 35 | 26 | 990 |
| 2031 | 1,193 | 86 | 37 | 27 | 1043 |
| 2032 | 1,202 | 86 | 37 | 28 | 1051 |
