# Supplementary material for: Economic evaluation of automated peritoneal dialysis among pediatric patients with end state kidney diseases in Thailand
Source: Sci Rep. 2025 May 25;15:18259. doi: 10.1038/s41598-025-00352-4 (PMC12104364; doi:10.1038/s41598-025-00352-4)
Supplement: Supplementary file 2 — Supplementary Material 2 [file 41598_2025_352_MOESM2_ESM.docx]

**Supplementary Table 2** Estimated number of ESKD pediatric patients requiring PD during 2016-2020

| **Year** | **Prevalent cases of ESKD across all age groups (A)** | **Estimated number of prevalent pediatric ESKD cases requiring PD**  **A*0.004*0.76** | **Incident cases of ESKD across all age groups (B)** | **Estimated number of incident pediatric ESKD cases requiring PD**  **B*0.004*0.76** |
| --- | --- | --- | --- | --- |
| 2016 | 100,970 | 310 | 24,439 | 75 |
| 2017 | 114,271 | 351 | 16,782 | 52 |
| 2018 | 128,987 | 396 | 15,094 | 46 |
| 2019 | 151,343 | 465 | 22,521 | 69 |
| 2020 | 170,774 | 524 | 19,772 | 61 |
