# Supplementary material for: Economic evaluation of automated peritoneal dialysis among pediatric patients with end state kidney diseases in Thailand
Source: Sci Rep. 2025 May 25;15:18259. doi: 10.1038/s41598-025-00352-4 (PMC12104364; doi:10.1038/s41598-025-00352-4)
Supplement: Supplementary file 3 — Supplementary Material 3 [file 41598_2025_352_MOESM3_ESM.docx]

**Supplementary Table 1** Model parameters

| **Parameters** | **Distribution** | **Mean** | **Standard error (SE)** | **Sources** |
| --- | --- | --- | --- | --- |
| **Epidemiological data** | | | | |
| Probability of APD to HD in year 1 | Beta | 0.0400 | 0.0040 | [14] |
| Probability of APD to HD in year 2 | Beta | 0.0100 | 0.0010 | [15] |
| Probability of APD to HD in year 3 | Beta | 0.0101 | 0.0010 |  |
| Probability of APD to HD in year 4 | Beta | 0.0204 | 0.0020 |  |
| Probability of APD to HD in year 5 | Beta | 0.0104 | 0.0010 |  |
| Probability of APD to HD in year 6 | Beta | 0.0316 | 0.0032 |  |
| Probability of APD to HD in year 7 | Beta | 0.0435 | 0.0043 |  |
| Probability of APD to HD in year 8 | Beta | 0.0455 | 0.0045 |  |
| Probability of APD to HD in year 9 | Beta | 0.0476 | 0.0048 |  |
| Probability of APD to HD in year 10 | Beta | 0.0500 | 0.0050 |  |
| Probability of PD to KT in year 1 | Beta | 0.0400 | 0.0040 | [14] |
| Probability of PD to KT in year 2 | Beta | 0.1667 | 0.0167 | [15] |
| Probability of PD to KT in year 3 | Beta | 0.1125 | 0.0113 |  |
| Probability of PD to KT in year 4 | Beta | 0.0845 | 0.0085 |  |
| Probability of PD to KT in year 5 | Beta | 0.0462 | 0.0046 |  |
| Probability of PD to KT in year 6 | Beta | 0.0484 | 0.0048 |  |
| Probability of PD to KT in year 7 | Beta | 0.0508 | 0.0051 |  |
| Probability of PD to KT in year 8 | Beta | 0.0536 | 0.0054 |  |
| Probability of PD to KT in year 9 | Beta | 0.0566 | 0.0057 |  |
| Probability of PD to KT in year 10 | Beta | 0.0600 | 0.0060 |  |
| Probability of APD to Death in year 1 | Beta | 0.0400 | 0.0040 | [14] |
| Probability of APD to Death in year 2 | Beta | 0.0104 | 0.0010 | [15] |
| Probability of APD to Death in year 3 | Beta | 0.0211 | 0.0021 |  |
| Probability of APD to Death in year 4 | Beta | 0.0215 | 0.0022 |  |
| Probability of APD to Death in year 5 | Beta | 0.0220 | 0.0022 |  |
| Probability of APD to Death in year 6 | Beta | 0.0225 | 0.0022 |  |
| Probability of APD to Death in year 7 | Beta | 0.0230 | 0.0023 |  |
| Probability of APD to Death in year 8 | Beta | 0.0235 | 0.0024 |  |
| Probability of APD to Death in year 9 | Beta | 0.0241 | 0.0024 |  |
| Probability of APD to Death in year 10 | Beta | 0.0247 | 0.0025 |  |
| Probability of CAPD to HD in year 1 | Beta | 0.0370 | 0.0037 | [14] |
| Probability of CAPD to HD in year 2 | Beta | 0.0100 | 0.0010 | [15] |
| Probability of CAPD to HD in year 3 | Beta | 0.0101 | 0.0010 |  |
| Probability of CAPD to HD in year 4 | Beta | 0.0204 | 0.0020 |  |
| Probability of CAPD to HD in year 5 | Beta | 0.0104 | 0.0010 |  |
| Probability of CAPD to HD in year 6 | Beta | 0.0316 | 0.0032 |  |
| Probability of CAPD to HD in year 7 | Beta | 0.0435 | 0.0043 |  |
| Probability of CAPD to HD in year 8 | Beta | 0.0455 | 0.0045 |  |
| Probability of CAPD to HD in year 9 | Beta | 0.0476 | 0.0048 |  |
| Probability of CAPD to HD in year 10 | Beta | 0.0500 | 0.0050 |  |
| Probability of CAPD to Death in year 1 | Beta | 0.0370 | 0.0037 | [14] |
| Probability of CAPD to Death in year 2 | Beta | 0.0104 | 0.0010 | [15] |
| Probability of CAPD to Death in year 3 | Beta | 0.0211 | 0.0021 |  |
| Probability of CAPD to Death in year 4 | Beta | 0.0215 | 0.0022 |  |
| Probability of CAPD to Death in year 5 | Beta | 0.0220 | 0.0022 |  |
| Probability of CAPD to Death in year 6 | Beta | 0.0225 | 0.0022 |  |
| Probability of CAPD to Death in year 7 | Beta | 0.0230 | 0.0023 |  |
| Probability of CAPD to Death in year 8 | Beta | 0.0235 | 0.0024 |  |
| Probability of CAPD to Death in year 9 | Beta | 0.0241 | 0.0024 |  |
| Probability of CAPD to Death in year 10 | Beta | 0.0247 | 0.0025 |  |
| Probability of HD to PD in year 1 | Beta | 0.3730 | 0.0373 | [14] |
| Probability of HD to PD in year 2 | Beta | 0.0415 | 0.0041 | [15] |
| Probability of HD to PD in year 3 | Beta | 0.0549 | 0.0055 |  |
| Probability of HD to PD in year 4 | Beta | 0.0352 | 0.0035 |  |
| Probability of HD to PD in year 5 | Beta | 0.0274 | 0.0027 |  |
| Probability of HD to PD in year 6 | Beta | 0.0019 | 0.0002 |  |
| Probability of HD to PD in year 7 | Beta | 0.0019 | 0.0002 |  |
| Probability of HD to PD in year 8 | Beta | 0.0019 | 0.0002 |  |
| Probability of HD to PD in year 9 | Beta | 0.0019 | 0.0002 |  |
| Probability of HD to PD in year 10 | Beta | 0.0454 | 0.0045 |  |
| Probability of HD to APD in year 1 | Beta | 0.3730 | 0.0373 | [14] |
| Probability of HD to APD in year 2 | Beta | 0.0415 | 0.0041 | [15] |
| Probability of HD to APD in year 3 | Beta | 0.0549 | 0.0055 |  |
| Probability of HD to APD in year 4 | Beta | 0.0352 | 0.0035 |  |
| Probability of HD to APD in year 5 | Beta | 0.0274 | 0.0027 |  |
| Probability of HD to APD in year 6 | Beta | 0.0019 | 0.0002 |  |
| Probability of HD to APD in year 7 | Beta | 0.0019 | 0.0002 |  |
| Probability of HD to APD in year 8 | Beta | 0.0019 | 0.0002 |  |
| Probability of HD to APD in year 9 | Beta | 0.0019 | 0.0002 |  |
| Probability of HD to APD in year 10 | Beta | 0.0454 | 0.0045 |  |
| Probability of HD to Death in year 1 | Beta | 0.0370 | 0.0037 | [14] |
| Probability of HD to Death in year 2 | Beta | 0.0104 | 0.0010 | [15] |
| Probability of HD to Death in year 3 | Beta | 0.0211 | 0.0021 |  |
| Probability of HD to Death in year 4 | Beta | 0.0215 | 0.0022 |  |
| Probability of HD to Death in year 5 | Beta | 0.0220 | 0.0022 |  |
| Probability of HD to Death in year 6 | Beta | 0.0225 | 0.0022 |  |
| Probability of HD to Death in year 7 | Beta | 0.0230 | 0.0023 |  |
| Probability of HD to Death in year 8 | Beta | 0.0235 | 0.0024 |  |
| Probability of HD to Death in year 9 | Beta | 0.0241 | 0.0024 |  |
| Probability of HD to Death in year 10 | Beta | 0.0247 | 0.0025 |  |
| Probability of KT to HD in year 1 | Beta | 0.0185 | 0.0019 | [16-17] |
| Probability of KT to HD in year 2 | Beta | 0.0066 | 0.0007 |  |
| Probability of KT to HD in year 3 | Beta | 0.0067 | 0.0007 |  |
| Probability of KT to HD in year 4 | Beta | 0.0207 | 0.0021 |  |
| Probability of KT to HD in year 5 | Beta | 0.0296 | 0.0030 |  |
| Probability of KT to HD in year 6 | Beta | 0.0316 | 0.0032 |  |
| Probability of KT to HD in year 7 | Beta | 0.0353 | 0.0035 |  |
| Probability of KT to HD in year 8 | Beta | 0.0351 | 0.0035 |  |
| Probability of KT to HD in year 9 | Beta | 0.0455 | 0.0046 |  |
| Probability of KT to HD in year 10 | Beta | 0.0538 | 0.0054 |  |
| Probability of KT to PD in year 1 | Beta | 0.0465 | 0.0047 | [16-18] |
| Probability of KT to PD in year 2 | Beta | 0.0165 | 0.0017 |  |
| Probability of KT to PD in year 3 | Beta | 0.0169 | 0.0017 |  |
| Probability of KT to PD in year 4 | Beta | 0.0519 | 0.0052 |  |
| Probability of KT to PD in year 5 | Beta | 0.0744 | 0.0074 |  |
| Probability of KT to PD in year 6 | Beta | 0.0794 | 0.0079 |  |
| Probability of KT to PD in year 7 | Beta | 0.0888 | 0.0089 |  |
| Probability of KT to PD in year 8 | Beta | 0.0883 | 0.0088 |  |
| Probability of KT to PD in year 9 | Beta | 0.1145 | 0.0114 |  |
| Probability of KT to PD in year 10 | Beta | 0.1353 | 0.0135 |  |
| Probability of KT to APD in year 1 | Beta | 0.0465 | 0.0047 | [16-18] |
| Probability of KT to APD in year 2 | Beta | 0.0165 | 0.0017 |  |
| Probability of KT to APD in year 3 | Beta | 0.0169 | 0.0017 |  |
| Probability of KT to APD in year 4 | Beta | 0.0519 | 0.0052 |  |
| Probability of KT to APD in year 5 | Beta | 0.0744 | 0.0074 |  |
| Probability of KT to APD in year 6 | Beta | 0.0794 | 0.0079 |  |
| Probability of KT to APD in year 7 | Beta | 0.0888 | 0.0089 |  |
| Probability of KT to APD in year 8 | Beta | 0.0883 | 0.0088 |  |
| Probability of KT to APD in year 9 | Beta | 0.1145 | 0.0114 |  |
| Probability of KT to APD in year 10 | Beta | 0.1353 | 0.0135 |  |
| Probability of KT to DEATH in year 1 | Beta | 0.0000 | 0.0000 | [16-18] |
| Probability of KT to DEATH in year 2 | Beta | 0.0076 | 0.0008 |  |
| Probability of KT to DEATH in year 3 | Beta | 0.0077 | 0.0008 |  |
| Probability of KT to DEATH in year 4 | Beta | 0.0116 | 0.0012 |  |
| Probability of KT to DEATH in year 5 | Beta | 0.0118 | 0.0012 |  |
| Probability of KT to DEATH in year 6 | Beta | 0.0059 | 0.0006 |  |
| Probability of KT to DEATH in year 7 | Beta | 0.0065 | 0.0007 |  |
| Probability of KT to DEATH in year 8 | Beta | 0.0263 | 0.0026 |  |
| Probability of KT to DEATH in year 9 | Beta | 0.0068 | 0.0007 |  |
| Probability of KT to DEATH in year 10 | Beta | 0.0063 | 0.0006 |  |
| **Cost data (baht per year)** | | | | |
| ***Direct medical costs*** |  |  |  |  |
| Cost of Tenckhoff catheter implantation | Gamma | 5,000 | 1,000 | Reimbursement |
| Cost of dialysis solution for CAPD including equipment | Gamma | 126 | 25 | [14] |
| Number of dialysis using CAPD per day | Gamma | 4 | 0.2470 | [14] |
| Cost of home visit and follow up | Gamma | 25,000 | 5,000 | Expert opinion |
| Cost of dialysis solution for APD including equipment | Gamma | 323 | 65 | [14] |
| Number of dialysis using APD per day | Gamma | 2 | 0.1597 | [14] |
| Cos of erythropoietin stimulating agent | Gamma | 7,405 | 590 | [14] |
| Cost of vascular access (AVF or AVG or Perm cath) HD set up | Gamma | 12,000 | 2,400 | Reimbursement |
| Cost of equipment for KT | Gamma | 355,579 | 79,717 | Reimbursement |
| Direct medical costs of OPD for APD | Gamma | 45,060 | 6,166 | [14] |
| Direct medical costs of IPD for APD | Gamma | 273,444 | 7,820 | [14] |
| Direct medical costs of OPD for CAPD | Gamma | 47,218 | 6,194 | [14] |
| Direct medical costs of IPD for CAPD | Gamma | 208,803 | 58,945 | [14] |
| Total direct medical costs for CAPD in year 1 | Gamma | 477,385 | 95,477 | [14] |
| Total direct medical costs of CAPD in year 2 | Gamma | 447,385 | 89,477 | [14] |
| Total direct medical costs of APD in year 1 | Gamma | 591,334 | 118,267 | [14] |
| Total direct medical costs of APD in year 2 | Gamma | 561,334 | 112,267 | [14] |
| Total direct medical costs of HD in year 1 | Gamma | 668,217 | 133,643 | [14] |
| Total direct medical costs of HD in year 2 | Gamma | 656,217 | 131,243 | [14] |
| Total direct medical costs of KT in year 1 | Gamma | 1,114,990 | 222,998 | [14] |
| Total direct medical costs of KT in year 2 | Gamma | 759,411 | 151,882 | [14] |
| ***Cost of transportation, accommodation, and food*** |  |  |  |  |
| Cost of transportation, accommodation, and food for CAPD | Gamma | 16,901 | 2,225 | [14] |
| Cost of transportation, accommodation, and food for APD | Gamma | 20,239 | 3,443 | [14] |
| Cost of transportation, accommodation, and food for HD | Gamma | 154,295 | 154,295 | [14] |
| Cost of transportation, accommodation, and food for KT | Gamma | 16,901 | 16,901 | [14] |
| ***Cost of caregivers and home renovation*** | | | | |
| Cost of caregivers for CAPD | Gamma | 71,020 | 15,076 | [14] |
| Cost of caregivers for APD | Gamma | 108,443 | 21,947 | [14] |
| Cost of home renovation for CAPD | Gamma | 28,863 | 5,257 | [14] |
| Cost of home renovation for APD | Gamma | 20,714 | 4,398 | [14] |
| Total cost of caregivers and home renovation for CAPD | Gamma | 99,884 | 16,980 | [14] |
| Total cost of caregivers and home renovation for APD | Gamma | 124,704 | 23,344 | [14] |
| Total costs of caregivers for HD | Gamma | 111,060 | 22,212 | [14] |
| Total costs of caregivers for KT | Gamma | 71,020 | 15,076 | [14] |
| **Direct non-medical costs** | | | | |
| Direct non-medical costs for CAPD | Gamma | 116,785 | 18,002 | [14] |
| Direct non-medical costs for APD | Gamma | 144,942 | 24,503 | [14] |
| Direct non-medical costs for HD | Gamma | 265,355 | 53,071 | [14] |
| Direct non-medical costs for KT | Gamma | 87,921 | 17,584 | [14] |
| **Utility** | | | | |
| Utility of CAPD | Beta | 0.8900 | 0.0445 | [22] |
| Utility of APD | Beta | 0.9400 | 0.0470 | [22] |
| Utility of HD | Beta | 0.8110 | 0.0406 | [23] |
| Utility of KT | Beta | 0.8300 | 0.0415 | [24] |
